# Advanced Drug Delivery Strategies in Geriatric Patients with Polypharmacy: Integrating Pharmacokinetics, Personalized Medicine, and Emerging Technologies

**DOI:** 10.3390/jcm15114359

**Published:** 2026-06-04

**Authors:** Dorota Bartusik-Aebisher, Katarzyna Bania, Blassan P. George, Klaudia Dynarowicz, David Aebisher

**Affiliations:** 1 Department of Biochemistry and General Chemistry, Faculty of Medicine, University of Rzeszów, 35-310 Rzeszów, Poland; dbartusikaebisher@ur.edu.pl (D.B.-A.); kdynarowicz@ur.edu.pl (K.D.); 2 English Division Science Club, Faculty of Medicine, University of Rzeszów, 35-310 Rzeszów, Poland; kb135595@stud.ur.edu.pl; 3 Laser Research Centre, Faculty of Health Sciences, University of Johannesburg, Doornfontein, P.O. Box 17011, Johannesburg 2028, South Africa; blassang@uj.ac.za; 4 Department of Photomedicine and Physical Chemistry, Faculty of Medicine, University of Rzeszów, 35-310 Rzeszów, Poland

**Keywords:** pharmacotherapy, polypharmacy, drug delivery systems, age-related physiological changes, deprescribing, dysphagia, artificial intelligence

## Abstract

**Background/Objectives**: The rapid growth of the global aging population, projected to reach 2.1 billion older adults by 2050, presents major challenges for pharmacotherapy and drug delivery. Age-related physiological changes affecting pharmacokinetics and pharmacodynamics, widespread polypharmacy, and functional impairments such as dysphagia, cognitive decline, and sensory or motor limitations reduce the effectiveness and safety of conventional “one-size-fits-all” medication approaches. This review aimed to evaluate the major barriers to effective drug delivery in older adults and to assess emerging patient-centered and technology-driven drug delivery systems designed to improve medication adherence, safety, and therapeutic outcomes in geriatric populations. **Methods**: A comprehensive narrative review of current literature was conducted focusing on geriatric pharmacotherapy, age-related barriers to medication administration, and advanced drug delivery technologies. The review analyzed evidence regarding modified oral formulations, transdermal systems, long-acting injectables, implantable devices, nanotechnology-based platforms, digital health integrations, pharmacogenomics, biomarker-guided therapy, and deprescribing strategies including STOPP/START criteria and Beers Criteria. Studies addressing polypharmacy, medication adherence, and personalized medicine in older adults were also evaluated. **Results**: Evidence indicates that older adults experience significant medication-related challenges due to multimorbidity, polypharmacy, and functional decline. Dysphagia affects more than half of nursing home residents, while polypharmacy prevalence reaches up to 86.6% in some populations. Emerging drug delivery technologies demonstrated potential to improve adherence, dosing precision, and patient convenience. Personalized approaches incorporating pharmacogenomics, biomarker-guided treatment, and AI-assisted dosing showed promise for optimizing therapy. However, major limitations remain, including underrepresentation of older adults in clinical trials, limited high-quality evidence supporting many polypharmacy interventions, and insufficient implementation of advanced drug delivery systems in routine clinical practice. **Conclusions**: Current evidence supports a transition from standardized medication approaches toward flexible, individualized, and patient-centered drug delivery strategies for older adults. Advanced delivery technologies and personalized pharmacotherapy may improve medication safety, adherence, and quality of life in aging populations, although stronger clinical evidence and broader implementation are still needed. Future progress will require interdisciplinary care models, improved geriatric representation in clinical research, and regulatory reforms supporting the integration of innovative drug delivery systems into routine healthcare practice.

## 1. Introduction

The 21st century is defined by an unparalleled global demographic transformation, characterized by the progressive aging of the world’s population. This shift, resulting from declining fertility rates and increasing life expectancy, is not a transient phenomenon but a fundamental restructuring of populations, with profound implications for healthcare systems, social policy, and the practice of medicine. The most recent global projections indicate that by 2030, one in six people worldwide will be aged 60 years or older, and by 2050, this demographic group will double to 2.1 billion, while the number of individuals aged 80 and over will triple to 426 million [1]. This represents a significant majority of older adults that will reshape the demand for and delivery of health services, including pharmacotherapy.

The scale of this demographic shift is further reflected in the relatively low prevalence of healthy aging among older adults worldwide. Meta-analyses indicate that a substantial proportion of aging individuals experience varying degrees of chronic disease, disability, or functional limitation, with successful aging rates declining progressively with advanced age [2,3]. These findings emphasize that increasing longevity does not necessarily correspond to prolonged healthspan, thereby intensifying the complexity of geriatric healthcare and pharmacotherapy.

As populations age, the prevalence of non-communicable diseases and multimorbidity continues to increase substantially, resulting in growing therapeutic complexity and medication burden among older adults. Reported prevalence estimates for multimorbidity and polypharmacy vary considerably across studies due to differences in population characteristics, healthcare settings, and operational definitions. Nevertheless, available evidence consistently demonstrates that older adults represent a population at particularly high risk for medication-related complications and complex pharmacotherapeutic management [4].

The challenge is particularly acute in low- and middle-income countries, which are entering a rapid demographic transition. Many of these healthcare systems remain insufficiently equipped with the infrastructure and long-term care services required to manage the growing aging population [1]. For example, in China, the burden of non-communicable diseases due to population aging indicates pronounced obstacles to healthcare delivery and long-term care services, requiring significant systematic adaptations [5]. The sheer number of older adults relying on chronic disease management necessitates a fundamental re-evaluation of therapeutic strategies. The data clearly indicate that the typical patient of the present and future is an older individual, often with multimorbidity and polypharmacy. This new demographic reality demands that drug delivery strategies move beyond a one-size-fits-all model and are instead optimized to meet complex physiological, functional, and social needs of the rapidly expanding global geriatric population [1,2,4].

This narrative review provides an integrated overview of current strategies aimed at optimizing drug delivery in older adults, with particular emphasis on the complex interaction between age-related physiological changes, multimorbidity, polypharmacy, and barriers affecting medication safety and adherence. The review discusses the impact of altered pharmacokinetics and pharmacodynamics on therapeutic outcomes in geriatric populations and evaluates both conventional and emerging drug-delivery approaches, including modified oral formulations, transdermal systems, long-acting injectable therapies, nanotechnology-based platforms, pharmacogenomic-guided dosing, and AI-assisted therapeutic optimization. Particular attention is given to personalized and interdisciplinary approaches designed to reduce medication burden, improve adherence, and enhance the safety and effectiveness of pharmacotherapy older adults. Additionally, the review highlights current translational challenges, implementation barriers, and future directions in geriatric drug-delivery research and clinical practice.

## 2. Materials and Methods

This article was conducted as a narrative review aimed at providing an integrated overview of current evidence regarding geriatric pharmacotherapy, polypharmacy, age-related physiological changes, and advanced drug-delivery strategies in older adults. Given the broad and interdisciplinary nature of the topic, the objective of the review was to critically synthesize clinically relevant, translational, and emerging evidence rather than perform a formal systematic review or meta-analysis.

A literature search was performed using PubMed and PubMed Central (PMC) databases to identify publications related to geriatric pharmacotherapy, polypharmacy, age-related pharmacokinetic and pharmacodynamic alterations, medication adherence, deprescribing, and advanced drug-delivery systems. Searches were conducted between January 2026 and May 2026, with the final search completed on 18 May 2026.

The search strategy combined Medical Subject Headings (MeSH) and free-text terms using Boolean operators. Representative search terms included: (“older adults” OR geriatric OR elderly) AND (polypharmacy OR deprescribing OR medication burden) AND (“drug delivery systems” OR transdermal OR “long-acting injectable” OR nanotechnology OR “orally disintegrating tablets” OR pharmacogenomics OR “medication adherence”).

Additional searches incorporated terms including:

- “dysphagia”,
- “cognitive impairment”,
- “frailty”,
- “personalized medicine”,
- “pharmacokinetics”,
- “pharmacodynamics”,
- “AI-assisted dosing”, and
- “geriatric drug delivery”.

Only articles published in English were considered. Eligible sources included systematic reviews, meta-analyses, randomized and non-randomized clinical studies, observational studies, translational and preclinical research, clinical guidelines, and landmark publications relevant to geriatric pharmacotherapy and drug-delivery optimization. Conference abstracts, duplicate publications, editorials without substantive scientific content, and studies not directly related to geriatric drug delivery or medication optimization were excluded.

Studies were evaluated for relevance to:

(1) age-related physiological changes affecting drug delivery,
(2) polypharmacy and medication-related burden in older adults,
(3) barriers to medication administration and adherence, and
(4) conventional and emerging drug-delivery technologies applicable to geriatric populations.

Particular emphasis was placed on studies providing mechanistic, clinical, translational, or implementation-related insight into geriatric drug delivery. “Up-to-date” literature was defined predominantly as studies published within the last 5–7 years, although landmark historical studies, consensus criteria (e.g., Beers Criteria, STOPP/START), and foundational geriatric pharmacology references were included where necessary for clinical and scientific context.

Due to the heterogeneity of available evidence, formal risk-of-bias assessment and quantitative meta-analysis were not performed. Instead, evidence was interpreted narratively with consideration of study design, sample size, healthcare setting, translational relevance, and methodological limitations. Greater emphasis was placed on systematic reviews, meta-analyses, and large cohort studies where available, while acknowledging that evidence supporting many emerging drug-delivery technologies remains preliminary, observational, translational, or preclinical.

## 3. Polypharmacy in Geriatrics

The global demographic shift toward an aging population, brings with it an inevitable and escalating clinical challenge: polypharmacy. Although recent studies continue to refine prevalence estimates and intervention strategies, the clinical concerns surrounding inappropriate medication use in older adults have been recognized for decades through landmark geriatric pharmacology frameworks, including the Beers Criteria and geriatric prescribing principles emphasizing individualized risk–benefit assessment [5]. Most commonly defined as the concurrent use of five or more medications, polypharmacy has emerged as one of the most pressing issues in geriatric pharmacotherapy. Its rate rises sharply with age, driven by increasing burden of multimorbidity and the following need for multiple disease-specific guidelines to be applied simultaneously to individual patients [4].

### 3.1. Epidemiology and Definitions

#### 3.1.1. Methodological Considerations

A 2025 cross-sectional study involving adults aged ≥60 years demonstrated that polypharmacy prevalence estimates vary considerably depending on whether medications are counted as active ingredients or unique products [6]. The findings suggest that conventional counting methods may substantially underestimate true medication burden, particularly among the oldest adults using combination therapies.

#### 3.1.2. Global Prevalence Estimates

A 2025 systematic review and meta-analysis of polypharmacy among older adults with diabetes reported a pooled prevalence of 59%, although prevalence estimates varied substantially across geographic regions and healthcare systems [7]. Lower prevalence rates were generally observed in high-income countries compared with Southern Europe and Asia.

Importantly, the extremely wide prediction interval suggests that polypharmacy prevalence estimates are highly dependent on study design, healthcare setting, population characteristics, and the operational definition of polypharmacy itself rather than reflecting a uniform global phenomenon. This methodological heterogeneity limits direct comparison between studies and highlights the need for standardized definitions in geriatric pharmacotherapy research [4,7].

Among community-dwelling older adults in China, meta-analytic evidence demonstrates substantial and regionally variable polypharmacy prevalence, with projections indicating continued growth over the coming decades [8]. These trends further emphasize the growing healthcare burden associated with medication management in rapidly aging populations.

#### 3.1.3. Prevalence Across Healthcare Settings

Institutionalized older adults consistently experience the highest burden of polypharmacy and hyperpolypharmacy across healthcare settings [9,10]. Evidence from nursing home populations and large systematic reviews indicates that extensive medication exposure is particularly common among frail older adults with multimorbidity and functional impairment.

Longitudinal cohort data further demonstrate that medication burden increases progressively prior to and following nursing home admission, particularly for psychotropic and cognitive-enhancing medications [10]. These findings suggest that transitions into institutional care are associated with increasing therapeutic complexity and escalating risk of medication-related complications.

#### 3.1.4. Hyperpolypharmacy and Future Projections

In nursing homes, extensive polypharmacy (≥10 medications) affects 18.4% of residents [9]. These numbers likely underestimate true burden, as they typically exclude over-the-counter and complementary products, which the Australian methodology study demonstrated can increase prevalence estimates by nearly 20 percentage points [6]. Projections from China indicate continued growth, with total polypharmacy cases expected to reach 131.7 million by 2035 [8], underscoring the urgent need for optimized drug delivery strategies.

### 3.2. Burden of Polypharmacy 

While the numerical definition of polypharmacy is useful for research and clinical screening, it captures only one dimension of a multifaceted problem. The concept of medication-related burden has gained increasing recognition as a more all-encompassing framework for understanding the impact of polypharmacy on older adults’ lives. This burden incorporates:

**Practical burden:** The physical and logistical challenges of managing multiple medications, including opening child-resistant containers, interpreting complex dosing schedules, administering different formulations such as injections, inhalers, and eye drops, and managing refills along with the costs [11].

**Psychological burden:** The emotional distress, anxiety, and sense of dependence associated with taking multiple daily medications, often serves as a constant reminder of illness and functional decline.

**Figure 1:**
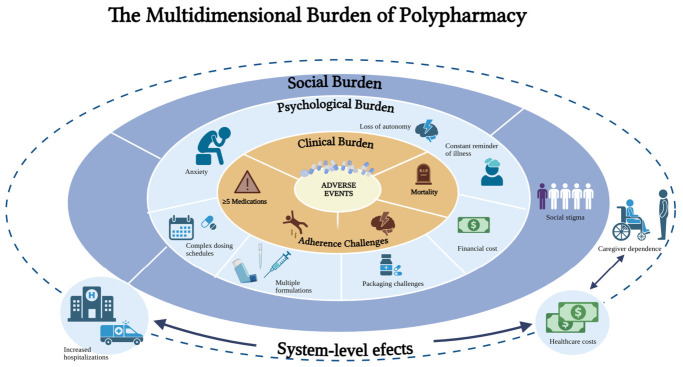
The multidimensional burden of polypharmacy in the geriatric population. Overview of the clinical, practical, psychological, social, and system-level burden associated with polypharmacy in older adults. Created in BioRender. Bania, K. (2026) https://BioRender.com/skkqwtz (accessed on 24 April 2026).

**Social burden:** The impact on social activities, travel, and daily routines since medication schedules must be accommodated, and the social labelling sometimes associated with visible medication use (Figure 1).

A qualitative meta-synthesis by Xin et al. (2025) examining older adults’ experiences with polypharmacy identified several recurring themes, including emotional distress related to long-term medication use, feelings of dependence, and the burden of managing complex medication regimens [12]. Medications were frequently described as a constant reminder of illness and aging, which contributed to anxiety and reduced sense of autonomy. In addition, the need to adhere to strict medication schedules was reported to disrupt daily routines as well as social activities. These findings highlight that polypharmacy is experienced not only as a clinical necessity but also as a significant psychological and social burden that can negatively affect quality of life.

### 3.3. Clinical Problems of Polypharmacy

The clinical problems of polypharmacy extend far beyond the obvious risks of adverse drug events. Although associations between polypharmacy and outcomes such as falls, hospitalization, cognitive decline, and mortality are consistently reported across observational studies, causality remains difficult to establish because polypharmacy frequently reflects underlying multimorbidity and frailty rather than acting as an isolated risk factor. Consequently, interpretation of these findings requires careful consideration of residual confounding and differences in study populations. An earlier systematic review of reviews done by Davies et al. (2020), synthesizing evidence across multiple studies in older adults, found consistent associations between polypharmacy and a range of negative outcomes, including increased risk of hospitalization, falls, and mortality [13].

**Adverse drug events and drug–drug interactions:** With each additional medication the risk of drug–drug interactions increases exponentially. A nationwide register study by Erhan, Wastesson, and Fastbom (2024) in Swedish older adults showed that drug duplications–an important contributor to drug–drug interactions and adverse drug events–remain prevalent despite some declining trends over time, highlighting the ongoing safety challenges associated with polypharmacy [14]. In real-world clinical and dispensing settings, potential drug–drug interactions are increasingly identified using electronic clinical decision-support system and pharmacy screening platform such as Micromedex^®^ (Merative, MI, USA). A retrospective observational study conducted in community pharmacy settings demonstrated substantial variability in the detection and classification of clinically relevant potential drug–drug interactions between different screening databases, emphasizing the importance of standardized medication review processes in older adults exposed to polypharmacy [15]. Emerging artificial intelligence-assisted systems may further support clinicians in identifying clinically significant interactions; however, concerns regarding accuracy, external validation, and clinical accountability remain particularly relevant in geriatric populations characterized by multimorbidity and complex medication regimens [16].

**Falls and fractures:** Polypharmacy is strongly associated with an increased risk of falls in older adults, particularly that involving psychotropic medications. This relationship has been consistently documented across both historical and contemporary geriatric literature, supporting the concept that medication-related harm represents a longstanding core issue in geriatric medicine rather than a newly emerging concern [17,18]. A systematic review and meta-analysis by Seppala et al. (2018), conducted by the EUGMS (European Geriatric Medicine Society) Task and Finish Group on fall-risk-increasing drugs, found that several classes of psychotropic medications (including antidepressants, antipsychotics, and sedatives) are significantly associated with elevated fall risk, underscoring the contribution of medication and specific drug classes to fall-related adverse outcomes [17].

**Cognitive impairment:** There is growing evidence which links medication burden (especially drugs with anticholinergic properties) to cognitive decline. A large case–control study conducted by Richardson et al. (2018) found that exposure to medications with strong anticholinergic effects was associated with and increased risk of dementia, with higher cumulative use linked to greater risk [19]. These findings highlight the role of specific medication classes within polypharmacy as potentially modifiable contributors to cognitive impairment in older adults.

**Frailty and functional decline:** Polypharmacy and frailty frequently coexist, with evidence suggesting that medication burden may contribute to the development of frailty over time. Longitudinal cohort study by Veronese et al. (2017) found that older adults exposed to polypharmacy had a significantly higher risk of developing frailty over an eight-year follow-up period, supporting the role of multiple medication use as an important predictor of functional decline in aging populations [20].

**Hospitalization and mortality:** The cumulative effects of polypharmacy contribute to increased healthcare utilization and poorer survival outcomes in older adults. A systematic review and meta-analysis by Li et al. (2022) found that polypharmacy is significantly associated with a higher risk of all-cause mortality, with evidence suggesting a dose–response relationship in which greater numbers of medications are linked to progressively increased risk [21].

### 3.4. Potentially Inappropriate Medications

A clinical dimension of polypharmacy is not solely the number of medications but their appropriateness for older patients. The concept of potentially inappropriate medications (PIMs) has been operationalized through several explicit criteria, most notably the Beers Criteria (updated 2023) and the STOPP/START criteria (Screening Tool of Older Persons’ Prescriptions/Screening Tool to Alert to Right Treatment). These tools are grounded in decades of geriatric pharmacotherapy research and remain foundational frameworks for medication review in older adults despite ongoing updates reflecting evolving evidence bases [22,23,24].

A systematic review and meta-analysis by Tian et al. (2023) including studies conducted worldwide from 17 countries, found that the pooled prevalence of PIM use among older adults is highly prevalent across care settings [25]. The analysis demonstrated substantial global variation with overall pooled prevalence of PMI use being 36.7% (95% CI, 33.4–40.0%), which indicated that a significant proportion of older individuals are exposed to medications for which the potential risks may outweigh the benefits or for which safer alternatives may be available.

Commonly implicated PIMs include long-acting benzodiazepines, first-generation antihistamines, nonsteroidal anti-inflammatory drugs, and medications with strong anticholinergic properties [22]. However, despite widespread use of tools such as the Beers Criteria and STOPP/START criteria, controversy remains regarding their universal applicability across healthcare systems and patient populations. A lot of recommendations are based predominantly on expert consensus and observational evidence rather than large randomized geriatric trials, reflecting the persistent underrepresentation of frail older adults in clinical research [22,23,25]. Cumulative exposure to anticholinergic medications has been linked to increased risk of dementia, cognitive decline, as well as other adverse outcomes, with higher overall anticholinergic burden associated with progressively greater risk [19].

### 3.5. The Prescribing Cascade and Its Consequences

Polypharmacy can create a self-reinforcing cycle through prescribing cascades, a phenomenon in which the adverse effect of one medication is mistaken for a new medical condition, which leads clinicians to prescribe additional medication rather than identify and manage the underlying problem. For instance, calcium channel blockers that are used to manage hypertension commonly cause peripheral edema, which may prompt the prescription of a diuretic. The diuretic can result in electrolyte imbalances or urinary symptoms, which in turn may lead to even more prescriptions, compounding medication burden and increasing the risk of adverse events.

Evidence from a population-based cohort study indicates that prescribing cascades contribute to a substantial proportion of a new prescriptions in older adults, estimated at around 15-20%. The most frequent initiators of these cascades are antihypertensives, psychotropic medications, and drugs with anticholinergic properties. These findings highlight the importance of careful medication review, ongoing monitoring, and consideration of non-pharmacological interventions to prevent unnecessary polypharmacy and mitigate its associated harms [26]. Patients with dementia face particularly complex medication management challenges and are disproportionately exposed to high-risk prescribing practices. Large population-based studies indicate that inappropriate or high-risk prescribing is substantially more common among individuals with dementia, particularly in those requiring functional assistance or institutional care [27].

**Figure 2:**
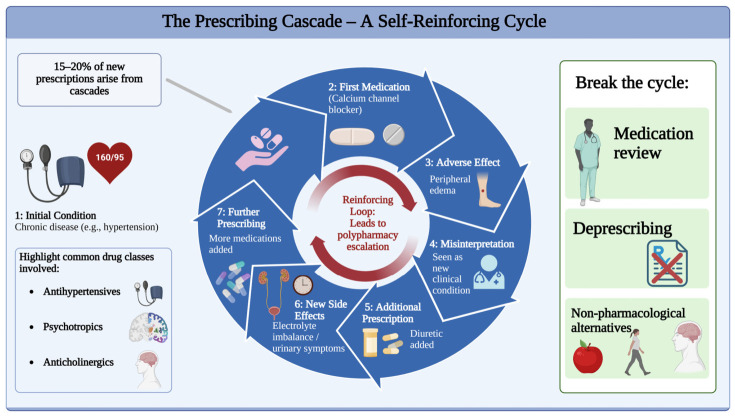
Schematic representation of the prescribing cascade. Schematic overview of the prescribing cascade and mechanisms contributing to progressive polypharmacy escalation in older adults. Created in BioRender. Bania, K. (2026) https://BioRender.com/7z6jt9r (accessed on 15 April 2026).

However, emerging evidence also suggests that the relationship between polypharmacy and cognitive outcomes may be more nuanced than medication count alone implies. In some populations with mild cognitive impairment, appropriate use of cardiovascular preventive therapies has been associated with reduced progression to dementia, highlighting the critical distinction between medication appropriateness and simple numerical definitions of polypharmacy [28]. These findings reinforce the importance of individualized pharmacotherapy rather than indiscriminate medication reduction in older adults. Prescribing cascades represent a specific and often underrecognized form of medication-related harm in which additional medications are prescribed to manage adverse effects caused by existing therapies (Figure 2). Population-based analyses have identified several clinically important prescribing cascades involving cardiovascular, psychotropic, and sedative medications in older adults [29]. These cascades may further exacerbate polypharmacy, increase therapeutic complexity, and contribute to avoidable adverse outcomes in geriatric pharmacotherapy.

### 3.6. Deprescribing and Medication Optimization

Deprescribing is a structured, supervised process of dose reduction or discontinuation of medications when potential harms outweigh expected clinical benefits within the context of an individual patient’s therapeutic goals, life expectancy, functional status, and overall burden of treatment. In geriatric populations, deprescribing has emerged as a critical component of medication optimization due to the high prevalence of multimorbidity, polypharmacy, frailty, and potentially inappropriate medication use. A structured roadmap for deprescribing in people with dementia addresses the unique challenges of living with dementia, including the variable disease course, high prevalence of distressing behavioral symptoms, and central role of care partners. Four key steps included in the deprescribing roadmap are: (1) identifying potential targets for deprescribing by eliciting medication-related goals and considering trade-offs; (2) developing a tapering plan; (3) completing additional actions necessary begore deprescribing; (4) providing close follow-up. Shared decision-making among this vulnerable population is supported by evidence-based strategies for communicating with patients and their care partners about deprescribing [30].

A systematic review of international clinical practice guidelines and expert tools addressing inappropriate chronic diuretic use and deprescribing identified 41 resources (14 guidelines, 27 tools) and synthesized 184 unique recommendations. Even so, the re-view found that deprescribing guidance was comparatively sparse and most often framed as prompts to consider stopping rather than practical how-to-pathways, with only 16 recommendations offering implementable support such as stepwise tapering or discontinuation, monitoring and safety-netting, or usable algorithms or flowcharts. This finding reinforces the pressing need for consolidated, implementation-ready deprescribing guidance to support safer long-term medication management and shared decision-making [31]. From the perspective of advanced frailty, dementia, or limited life expectancy, deprescribing preventative medications requires particular attention. Shared decision-making approaches that respect patient preferences and goals of care are part of the carful considerations needed to be taken into account when evaluating the risks and benefits of continuing versus stopping preventative therapies in these vulnerable populations [32].

### 3.7. Caregiver and Patient Perspectives

Understanding the lived experience of polypharmacy is essential for developing patient-centered interventions. A recent qualitative study using focus group interviews with older adults, their relatives, and healthcare professionals explored how multiple medications affect daily life and care practices. Participants described wide variability in how they experience polypharmacy–from challenges remembering complex regimens and unintentional or intentional non-adherence, to frustrations with generic substitutions and the burden of organizing multiple medicines. Difficulties in communicating about medications, coordinating schedules, and maintaining accurate medication lists presented as a barrier to safe and effective use. Family members and caregivers frequently played an active role in supporting medication reviews and collaborative discussions. These insights underscore that addressing polypharmacy requires attention not only to describing and medication optimization but also to the practical, relational, and communication challenges older adults and their caregivers face in medication use [33]. Critical for successful implementation of optimized drug delivery is effective patient and caregiver education. A stakeholder-informed review process identified practical strategies for improvement of medication management that directly addressed the needs of family caregivers and home health professionals. Focusing on addressing dementia-specific challenges, clarifying medication management tasks, reducing role ambiguity, and establishing care transition quality measurement and feedback mechanisms [34].

Stakeholder priorities should guide the development of patient education materials. Research identifying priority areas for medication management information resources for people with dementia and their carers reinforces the need for question prompts to facilitate partnership between individuals, carers, and health professionals; informed consent and active participation in shared decision-making for prescribed medications; and exploring solutions in order to address medication challenges such as adherence, progressive behavioral changes, transitions in care, and cultural considerations [35].

The integration of digital health interventions for polypharmacy management in older adults has been systematically evaluated using the Non-adoption, Abandonment, Scale-up, Spread, and Sustainability (NASSS) framework. Successful adoption of technology-supported interventions was associated with user-centered technology design, strong clinical leadership, workflow integration, and organizational readiness. Interventions incorporating multidisciplinary collaboration, continuous feedback, and iterative adaptation showed stronger sustainability, while common barriers included technological complexity, poor interoperability, lack of provider engagement, insufficient training, and regulatory constraints. Decision tree analysis identified usability, early stakeholder engagement, organizational alignment, and system adaptability as key factors predicting successful adoption and sustainability [36].

## 4. Age-Related Physiological Changes Impacting Drug Delivery

The drug handling and response in older adults is fundamentally reshaped by the progressive physiological alterations induced by the aging process. Due to those changes not only all phases of pharmacokinetics are affected (absorption, distribution, metabolism, and excretion) but also pharmacodynamic sensitivity to many medications [37,38]. Additionally, geriatric syndromes such as frailty and sarcopenia contribute further interindividual variability, complicating drug delivery optimization in this population [38,39]. It is essential to understand these age-related changes to design and select appropriate drug delivery systems that accommodate the unique needs of older adults.

### 4.1. Pharmacokinetic Alterations

Age-related physiological changes significantly alter drug absorption, distribution, metabolism, and excretion (ADME), collectively influencing pharmacokinetics in older adults [38,39]. These pharmacokinetic principles have long formed the basis of geriatric prescribing theory and remain central to modern personalized pharmacotherapy approaches [40].

#### 4.1.1. Absorption

Gastrointestinal changes related to ageing include reduced gastric acidity, delayed gastric emptying, decreased splanchnic blood flow, and diminished intestinal surface area [38,39]. While passive diffusion remains largely unaffected, drugs that require active transport (e.g., iron, calcium) may show reduced absorption [41]. Reduced first-pass metabolism may have diminished bioavailability in prodrugs requiring hepatic activation [38].

#### 4.1.2. Distribution

Aging alters body composition, resulting in a 20–40% increase in adipose tissue and a 10–15% decrease in lean body mass and total body water, significantly influencing drug distribution kinetics and volume of distribution [40,42]. Consequently, lipophilic drugs (e.g., diazepam, amiodarone) exhibit increased volume of distribution and prolonged elimination half-lives, while hydrophilic drugs (e.g., digoxin, lithium) show reduced distribution volume with higher plasma concentrations [41,43]. Along with age plasma albumin levels decline, particularly in acutely ill or malnourished patients, increasing free fractions of highly protein-bound drugs (e.g., warfarin, phenytoin) [37,39].

#### 4.1.3. Metabolism

Hepatic metabolism undergoes significant age-related changes. Liver mass and hepatic blood flow decrease by approximately 0.5–1.5% per year after age 40, reducing first-pass clearance [38,39]. Phase I metabolism mediated by cytochrome P450 (CYP) enzymes–particularly CYP3A4, CYP2C19, and CYP2D6–declines with age, whereas phase II conjugation reactions (e.g., glucuronidation) are relatively preserved [37]. Transcriptomic analysis across 52 human tissues revealed consistent age-related declines CYP enzymes, contrasting with increased UDP-glucuronosyltransferase expression in digestive and urinary systems [44].

#### 4.1.4. Excretion

Renal excretion is the most clinically significant pharmacokinetic change in older adults. Glomerular filtration rate declines by approximately 1mL/min per year after age 40, even in the absence of overt kidney disease [38,41]. Creatinine clearance–nor serum creatinine alone–must guide dosing of renally eliminated drugs. Age-related declines in tubular secretion and reduced renal blood flow further impair drug excretion [37,38,45].

Collectively, age-related physiological changes substantially alter drug absorption, distribution, metabolism, and excretion, thereby increasing interindividual variability in therapeutic response and vulnerability to adverse drug events in older adults [42,46]. The major pharmacokinetic alterations associated with aging and their therapeutic implications are summarized in Table 1.

These pharmacokinetic alterations contribute substantially to the increased complexity of medication management in geriatric populations and reinforce the importance of individualized dosing strategies and careful therapeutic monitoring.

### 4.2. Pharmacodynamic Changes

**Figure 3:**
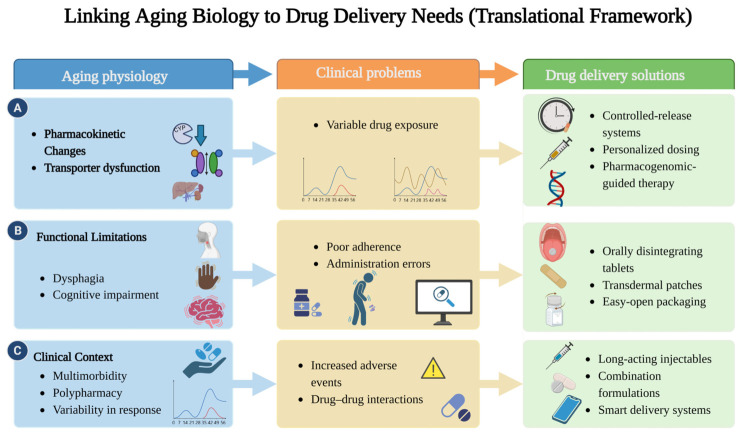
The interface between geriatric biology and drug delivery innovation. Translational framework linking age-related physiological changes with clinical challenges and targeted drug-delivery strategies in older adults. Created in BioRender. Bania, K. (2026) https://BioRender.com/b9r1ac9 (accessed on 3 April 2026).

The relationship between drug concentration at the site of action and the resulting pharmacological effect (essentially what the drug does to the body) is referred to as pharmacodynamics [39]. In older adults, age-related alterations in receptor density, receptor affinity, post-receptor signalling pathways, and homeostatic compensatory mechanisms can lead to enhanced or diminished drug responses independent of pharmacokinetic changes (Figure 3) [38,47].

#### 4.2.1. Receptor-Level Changes

Neuroimaging studies have demonstrated age-dependent changes in key neurotransmitter receptor systems. Rzeczycki et al. (2025) report alterations in the density and sensitivity of serotonergic, dopaminergic, and adrenergic receptors in the aging brain, contributing to altered responses to psychotropic medications [37]. These molecular changes help explain why older adults are prone to experience exaggerated effects or adverse reactions at standard therapeutic doses.

#### 4.2.2. Enhanced Sensitivity to Specific Drug Classes

Older adults exhibit particularly pronounced sensitivity to certain drug classes:

− Anticholinergic medications: The anticholinergic burden represents a significant iatrogenic concern in older patients. As highlighted by Tadic et al. (2025), anticholinergic medicines contribute notably to prescribing cascades and inappropriate polypharmacy, with the BEERS criteria identifying anticholinergic burden as a major safety issue since 1991 [48]. Older adults–especially those with cognitive impairment–are particularly vulnerable to central nervous system effects such as drowsiness, confusion, and delirium [37,48].
− Benzodiazepines and sedatives: The increased sensitivity results from age-related changes in GABA (Gamma-Aminobutyric Acid) receptor function and impaired homeostatic responses [37,38].
− Antihypertensive agents: A prospective study by Hassan et al. (2025) that compares perindopril pharmacokinetics and pharmacodynamics between younger (<50 years) and older (>70 years) patients found that older adults showed significantly higher exposure to the active metabolite perindoprilat, alongside a trend toward greater systolic blood pressure reduction [49]. Along with the age decreases baroreceptor reflex sensitivity, which further increases susceptibility to orthostatic hypotension and falls with antihypertensive medications [38].
− Anesthetic agents: Coetzee et al. (2025) emphasize that for most anesthetic drugs, older adults require lower doses to achieve the same plasma concentrations, and at any given plasma and effect-site concentration, they will have more profound clinical effects than younger patients [47]. The authors argue for the “start low, go slow” principle along with the close monitoring.

#### 4.2.3. Reduced Sensitivity to Other Drug Classes

Conversely, some drug classes demonstrate diminished side effects in older adults. The efficacy of beta-blockers and beta-agonists is potentially reduced due to declining of beta-adrenergic receptor responsiveness caused by age. Similarly, reduced baroreceptor sensitivity may diminish compensatory responses to vasodilators [38,47].

#### 4.2.4. Role of Frailty and Homeostatic Decline

A state of increased vulnerability to stressors–commonly referred to as frailty–significantly impacts pharmacodynamic responses. A systematic review by Gutiérrez-Valencia et al. (2024) examining the relationship between frailty and adverse drug reactions in older adults found that frailty was consistently associated with a higher risk of adverse drug reactions across multiple studies, with frail older adults demonstrating reduced physiological reserve to compensate for drug-induced perturbations [50]. Due to this diminished compensatory capacity the risk of adverse drug events, including falls, delirium, and hypotension increases, even with appropriately dosed medications [38,50].

#### 4.2.5. Clinical Implications

Understanding age-related pharmacodynamic changes is essential for safe prescribing in older adults. Standard “one-size-fits-all” dosing regimens often fail to account for increased sensitivity to certain drugs while potentially underdosing others [37,47]. To optimize therapeutic outcomes while minimizing harm, individualized approaches that consider frailty status, cognitive function, and comorbidity burden are necessary [37,38,50].

### 4.3. Frailty and Sarcopenia

Drug delivery and pharmacotherapy outcomes in older adults are severely impacted by overlapping geriatric syndromes such as frailty and sarcopenia. Frailty is a state of increased vulnerability to stressors resulting from age-related declines in physiological reserve across multiple organ systems [51]. Sarcopenia, defined as the progressive loss of skeletal muscle mass, strength, and function, affects drug metabolism, distribution, and physical capacity for medication self-administration [52].

#### 4.3.1. Epidemiology and Clinical Significance

Sarcopenia affects approximately 10% of individuals aged over 60 years, with prevalence increasing substantially with age. Increased risk of falls, fractures, hospitalization, and mortality, contributes to the loss of independence in older adults [53]. Depending on the population studied and frailty criteria employed, the frailty prevalence among older adults varied widely, ranging from 0.9% to 89.2% [52].

#### 4.3.2. Frailty, Polypharmacy

**Figure 4:**
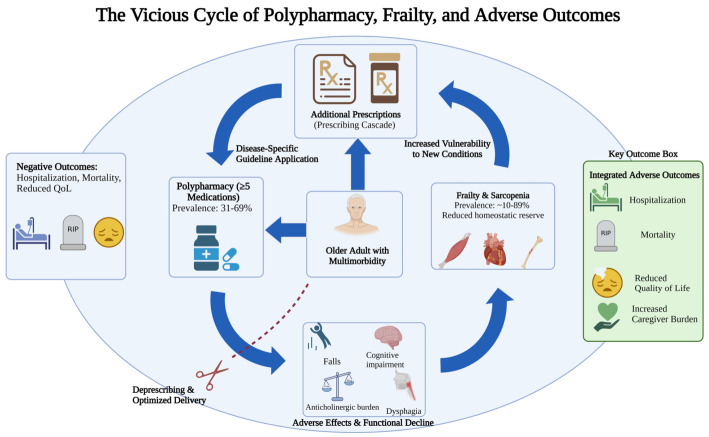
The Vicious Cycle of Polypharmacy, Frailty, and Adverse Outcomes. Conceptual model illustrating the self-reinforcing interaction between polypharmacy, frailty, prescribing cascades, and adverse clinical outcomes in older adults. Created in BioRender. Bania, K. (2026) https://BioRender.com/w52vo5c (accessed on 2 May 2026).

The relationship between frailty and medication use is complex and bidirectional. A scoping review by Sharma et al. (2025) examining medication use in older adults with frailty found that polypharmacy (5–9 medications) and hyper-polypharmacy (above 10 medications) were notably more common among those with frailty, with polypharmacy rates ranging from 1.3% to 96.4% across studies [52]. The review also reported that potentially inappropriate medication (PIM) prevalence among individuals with varying levels of frailty ranged from 2.4% to 95.9%, underscoring the substantial medication burden in this vulnerable population (Figure 4).

#### 4.3.3. Impact on Drug Pharmacokinetics and Safety

The ability to compensate for drug-induced physiological perturbations is impaired by the reduced homeostatic reserve characteristic of frailty. Rodriguez-Espeso et al. (2025) identify polypharmacy, multimorbidity, frailty, and age-related pharmacokinetic and pharmacodynamic changes as key risk factors associated with higher rates of adverse drug reactions in older adults [54]. As emphasized by authors, medication review and deprescribing should be performed in conjunction with a comprehensive geriatric assessment.

#### 4.3.4. Sarcopenia and Drug Delivery Challenges

Practical aspects of drug delivery are directly impacted by sarcopenia. Reduced muscle mass affects the reliability of intramuscular injection sites and may alter absorption from depot formulations. In addition, core components of sarcopenia assessment such as diminished hand grip strength and motor function compromise the ability to open medication packaging, manipulate oral solid dosage forms, and self-administer injections [41].

#### 4.3.5. Supporting Self-Management in Frail Older Adults

To address the challenges faced by frail older adults in managing multiple medicines, we require targeted interventions. An experience-based-co-design study by Previdoli et al. (2025) developed a complex intervention to support medicines self-management for older people living with frailty and polypharmacy [55]. Five key areas have been addressed: (1) knowledge of medicines and checking medicines received; (2) organizing medicines supply; (3) adherence and self-monitoring; (4) dealing with changes in medicines; and (5) knowledge of help available and how to seek it.

#### 4.3.6. Implications of Drug Development and Clinical Practice

The recognition of sarcopenia and frailty as critical determinants of drug response has important implications of geriatric pharmacotherapy. For frail and sarcopenic populations traditional dosing strategies may be based on chronological age or body weight may be inadequate. Some of the emerging approaches include the integration of frailty assessment into therapeutic drug monitoring protocols, pharmacist-led deprescribing interventions using validated tools such as STOPPFrail, and the development of drug delivery systems designed specifically for patients with functional limitations [52,54,55].

## 5. Barriers and Clinical Impact of Effective Drug Delivery in Geriatric Patients 

The effective delivery of medications to older adults is hindered by a complex interplay of patient-related, medication-related, and system-level barriers. These obstacles contribute to medication errors, non-adherence, adverse drug events, and suboptimal therapeutic outcomes in geriatric populations [37,38].

### 5.1. Dysphagia

#### 5.1.1. Epidemiology and Clinical Consequences

Dysphagia is one of the most clinically significant barriers to effective oral medication administration in older adults. Impaired swallowing complicates the safe administration of oral solid medication forms and is associated with adverse outcomes such as malnutrition, dehydration, aspiration pneumonia, functional decline, and increased mortality [56,57]. The prevalence of dysphagia varies depending on the healthcare setting and assessment methodology, with estimates ranging from approximately 9–23% among community-dwelling older adults, and significantly higher rates observed in institutionalized and nursing home populations [57,58,59,60]. Moderate to severe swallowing impairment is particularly common in the oldest-old and most vulnerable individuals.

#### 5.1.2. Medication-Related and Functional Factors

The pathophysiology of dysphagia in aging is multifactorial and reflects the interaction between age-related physiological decline, neurodegenerative processes, frailty, sarcopenia, and medication-related factors. Recent neurogeriatric studies highlight the impact of presbyphagia, impaired pharyngeal sensation, reduced neuroplasticity, and age-related muscle dysfunction on swallowing disorders [56]. Increased exposure to anticholinergic medications is associated with swallowing impairment, new onset dysphagia during hospitalization, and salivary flow disorders, further illustrating the complex relationship between polypharmacy and functional decline in geriatric populations [61].

Dysphagia significantly complicates adherence in both community and institutional settings. Caregivers frequently report challenges related to inappropriate dosage forms, insufficient guidance on medication modifications, financial barriers, and fragmented communication between healthcare professionals [62]. These findings underscore the need for interdisciplinary treatment strategies that integrate physicians, pharmacists, nurses, speech-language pathologists, patients, and caregivers.

#### 5.1.3. Clinical and Therapeutic Implications

Several therapeutic and technological approaches can improve medication administration for patients with swallowing disorders. Modified oral formulations, such as orally disintegrating tablets, oral films, liquid formulations, and alternative extraoral delivery systems, can improve adherence and reduce the risk of aspiration. New technologies, including closed-system medication delivery devices designed to safely crush and dissolve oral medications, can further improve access to treatment while reducing the risk of occupational exposure for healthcare workers [63]. Collectively, these approaches demonstrate the importance of individualized medication administration strategies tailored to the functional limitations of older adults.

### 5.2. Cognitive Impairment

Cognitive impairment represents a major barrier to effective drug delivery in geriatric patients due to its effect on medication adherence, self-management capacity, and safety. The complex and bidirectional relationship between cognitive decline and medication management involves polypharmacy, anticholinergic burden, and the progressive loss of functional abilities necessary for independent medication administration [64,65].

#### 5.2.1. Prevalence and Impact on Adherence

Cognitive impairment substantially compromises medication adherence in older adults. A large systematic review and meta-analysis demonstrated that cognitive deficits and frailty are consistently associated with reduced adherence rates across geriatric populations [64]. These findings emphasize the major influence of cognitive and functional decline on therapeutic effectiveness and long-term medication management in older adults.

The relationship between medication burden and cognitive function has been further supported by studies demonstrating significant associations between polypharmacy, anticholinergic exposure, and subjective memory complaints in community-dwelling older adults [65,66]. These findings suggest that medication-related cognitive effects may contribute to early functional decline and impaired self-management capacity in geriatric populations.

#### 5.2.2. Memory Aids and Technology-Based Solutions

The use of cognitive aids and technology may help attenuate medication management challenges in older adults with cognitive impairment. In their study Fujita et al. (2026) compare medication management behaviours in healthy older adults and patients with Alzheimer’s disease using Internet of Things devices and they discovered that operational errors occurred in both groups [67]. Factors influencing successful medication management included comprehension of spoken language and prospective memory-common to both groups-while disorientation and attention deficits were specific factors affecting the group with Alzheimer’s disease.

A stakeholder-informed review by Cai et al. (2025) identified practical strategies for improving medication management during care transitions for older adults with dementia [34]. Among the top strategies were: (1) secure storage of home medications in order to decrease risk of adverse events; (2) usage of cognitive aids for medication storage and administration to reduce error risk; and (3) providing dementia-specific training to home health staff.

#### 5.2.3. Caregiver Involvement and Support

Caregiver involvement plays an important role in improving medication adherence and therapeutic outcomes among cognitively impaired older adults. Systematic review evidence indicates that caregiver-supported medication management is associated with substantially higher adherence rates, particularly in patients with dementia and severe cognitive impairment [64].

Additional studies examining dietary supplement administration in individuals with dementia further highlight the complexity of medication management in cognitively vulnerable populations. Caregivers frequently assist with medication and supplement administration; however, concerns regarding administration errors, supplement safety, and inadequate guidance from healthcare professionals remain common [68]. These findings emphasize the importance of caregiver education, interdisciplinary communication, and structured medication support systems in geriatric care.

#### 5.2.4. Stakeholder Priorities for Medication Management Resources

By using community-based participatory research, an exploratory study by Watson et al. (2025) identified four priority areas for medication management information resources for people with dementia and their carers: (1) question prompts to facilitate partnership between individuals, carers, and health professionals; (2) informed consent and active participation in shared decision-making; (3) additional information on the benefits and risks of common medications; and (4) exploring solutions to address medication challenges (e.g., adherence, progressive behavioural changes, transitions in care, and cultural considerations) [35].

#### 5.2.5. Clinical Implications

Multifaceted approaches such as routine cognitive screening in older adults, minimizing anticholinergic, utilizing memory aids and simplified regimens, engaging caregivers in medication management, and providing dementia-specific training to healthcare professionals are required when addressing cognitive impairment as a barrier to drug delivery [34,64,66]. The distinction between medication quantity and medication appropriateness—especially the potential beliefs of certain preventative medications—must be carefully considered in treatment planning for cognitively impaired older adults [28].

### 5.3. Sensory and Motor Limitations

Sensory impairments—specifically vision and hearing loss—and motor limitations are frequent among older adults and create substantial barriers to safe and effective medication self-administration. Due to those age-related functional obstacles medication adherence can be compromised, error risk can be increased, and therapeutic outcome is ultimately impacted [41,69].

#### 5.3.1. Visual Impairment

Vision impairment substantially complicates medication management in older adults and may contribute to medication administration errors, dosing inaccuracies, and reduced treatment adherence. Studies involving visually impaired populations demonstrate considerable difficulties with identifying medications, distinguishing drug packaging, measuring liquid formulations, and verifying expiration dates. Older adults with multimorbidity, polypharmacy, lower educational status, or severe visual impairment appear particularly vulnerable to medication-related difficulties and frequently require caregiver or external assistance for safe medication administration [69].

A qualitative interview study by Morrison et al. (2026) with independent prescribers in primary care explored prescribing challenges for older people with sensory impairment, identifying key barriers in clinical practice [70]. A related scoping review conducted by the same author in 2025 examined guidelines and resources to promote evidence-based prescribing for this population [69].

#### 5.3.2. Hearing Impairment

Age-related hearing loss (ARHL) is the most common sensory deficit among older adults that affects about 40% of those over age 65. ARHL begins around age 50 and has a profound effect on emotional, social, and physical aspects of everyday life. Lack of treatment for this condition has been found to lead to cognitive decline, social isolation, and adverse physical effects, such as loss of balance, which can result in falls [71].

Exploring of communication barriers in community pharmacy settings identified that background noise reduced the confidentiality and effectiveness of communication between pharmacists and older adults suffering from hearing loss. Due to embarrassment, pharmacy clients expressed reluctance to disclose their hearing needs even though pharmacists emphasized a need to know about hearing loss in order to adapt communication style effectively [72].

Cognitive decline, depression, increased risk of falls, and decreased quality of life are only few of the challenges that are associated with ARLH. ARLH remains underdiagnosed and undertreated despite its high prevalence, which is partly due to its gradual onset, stigma, and lack of standardized screening protocols. In order to improve early detection and management, it is recommended that adults aged 50 and above do a routine screening [73].

#### 5.3.3. Motor Limitations

Motor limitations include reduced grip strength, fine motor control, and range of motion and directly impact the ability to manipulate medication packaging, open child-restraint containers, handle small tablets, and self-administer injections or eye drops. They are particularly common in elder patients diagnosed with conditions such as arthritis, Parkinson’s disease, or post-stroke deficits. The need for user-friendly packaging and alternative drug delivery systems (e.g., transdermal patches, pre-filled syringes) is well-recognized in geriatric pharmacotherapy [41].

#### 5.3.4. Combined Impact of Multiple Impairments

Oftentimes older adults experience sensory and motor impairments simultaneously, which adds to the challenges of managing medications. A scoping review by Asante et al. (2025) examining assessment tools for medication self-management capacity in community-dwelling older adults suffering from sensory impairment identified the need for validated instruments to evaluate functional capacity for independent medication use [74]. The authors emphasize that healthcare professionals are in need of training to assess and address the specific needs of patients with combined impairments.

#### 5.3.5. Clinical Implications

Multifaceted approaches are required to address the sensory and motor limitations as barriers to drug delivery, those including: (1) routine screening for vision, hearing, and motor function in older adults; (2) providing of accessible medication information (large print, braille, audio, or digital formants); (3) use of assistive technologies including hearing loops, amplified phones, and talking prescription devices; (4) pharmacist-led education and counselling adapted to patients’ sensory abilities; (5) consideration of alternative drug delivery systems when standard oral formulations prove challenging; and (6) caregiver involvement when independent self-administration is not feasible [72,73,75].

### 5.4. Drug–Drug Interaction and Intervention Strategy 

Polypharmacy and drug–drug interactions (DDIs) represent interconnected barriers to effective drug delivery in geriatric patients, creating a complex web of medication-related risks that disproportionately affect older adults [76,77].

#### 5.4.1. Prevalence and Clinical Significance

Polypharmacy is highly prevalent among older adults and represents one of the primary drivers of clinically significant drug–drug interactions (DDIs). Interactions involving anticholinergic and sedative medications are particularly common and have been associated with frailty, sarcopenia, and impaired physical functioning in community-dwelling geriatric populations [76]. These findings highlight the close relationship between functional decline, and increased vulnerability to adverse drug events in older adults.

#### 5.4.2. Mechanisms and Risk Factors

The interrelationship between polypharmacy, multimorbidity, and DDIs creates a high-risk environment for medication-related harm. As reported in the review examining pharmacotherapy in older persons the risks associated with polypharmacy are escalated due to age-related physiological changes (e.g., reduced hepatic and renal function, decreased cytochrome P450 enzyme activity, and altered body composition). Drug interactions represent one of the most preventable medical errors in older persons’ pharmacotherapy, yet they remain underrecognized in clinical practice [77].

#### 5.4.3. Intervention Strategies

A 2025 review of polypharmacy interventions in older adults notes that medication reviews using STOPP/START, Beers Criteria, and the Medication Appropriateness Index improve prescribing quality. High-intensity interventions involving multidisciplinary teams demonstrate potential to reduce hospitalizations and adverse drug reactions. Shared decision-making, digital deprescribing tools, and artificial intelligence-driven (AI) clinical decision support systems are part of the emerging strategies that may enhance polypharmacy management. However, variability in intervention intensity and poor implementation strategies contribute to inconsistent finding across studies [78,79].

Importantly, the current evidence base remains limited by substantial methodological heterogeneity, including differences in intervention components, duration of follow-up, outcome measures, and healthcare settings. Even though multidisciplinary deprescribing interventions frequently improve prescribing appropriateness, evidence for consistent reductions in mortality or long-term hospitalization still remains comparatively weak [79].

#### 5.4.4. Clinical Implications

In order to address polypharmacy and DDIs in geriatric patients, we require systemic approaches such as routine medication reconciliation, use of validated DDI screening tools, regular deprescribing of unnecessary medications, and interdisciplinary collaboration between prescribers and pharmacists [77,79].

### 5.5. Socioeconomic and Caregiver Factors

Socioeconomic status (SES) and caregiver availability represent critical yet often underrecognized barriers to effective drug delivery in geriatric patients. These factors influence medication access, adherence, health literacy, and the capacity for safe medication self-management [80,81].

#### 5.5.1. Socioeconomic Barriers

Lower socioeconomic status (SES) is consistently associated with poorer medication-related outcomes in older adults. Individuals with lower SES are more likely to report reduced satisfaction with medications, lower health literacy, poorer self-reported health, and greater interest in deprescribing interventions compared with older adults of higher socioeconomic status [80]. These findings suggest that socioeconomic inequalities substantially influence medication experiences, therapeutic engagement, and healthcare communication in geriatric populations.

Medication affordability remains an important barrier to adherence among older adults with chronic disease. Financial limitations, insufficient family support, and inadequate disease understanding have all been identified as contributors to suboptimal medication adherence in geriatric populations [82]. These barriers may disproportionately affect socially vulnerable individuals with complex therapeutic regimens.

#### 5.5.2. Heath Literacy and Digital Literacy

Older adults with lower SES are disproportionately affected by limited health literacy, which significantly influences medication adherence and self-management capacity. Meta-analytic evidence demonstrates that structured health literacy interventions can improve medication adherence and self-efficacy among older adults with chronic disease [81]. These findings support the integration of targeted educational and communication strategies into routine geriatric care.

eHealth literacy, defined as the ability to access and apply digital health information, is also associated with medication beliefs and adherence behaviors in older adults with chronic disease. Higher eHealth literacy levels have been linked to improved medication adherence, whereas concerns regarding medication safety and adverse effects may negatively influence adherence behaviors [83]. These findings highlight the growing importance of digital health competencies in contemporary geriatric pharmacotherapy.

#### 5.5.3. Caregiver Factors

Caregiver involvement significantly improves medication adherence and therapeutic outcomes in cognitively vulnerable older adults. Systematic review evidence indicates that caregiver-supported medication management is particularly beneficial in populations with dementia and severe psychiatric disease [64]. Conversely, insufficient family or social support may contribute to suboptimal adherence and reduced treatment effectiveness [82]. These findings reinforce the importance of caregiver engagement and interdisciplinary support in geriatric medication management.

A 2025 cross-sectional study of 106 home care patients aged 65 years or older taking at least one high-risk medication reported a median use of 8 medications per patient, of which 2 were high-risk medications. Most patients were supported by home care nurses for medication preparation, and a mean of 5 potential adverse drug reactions were reported per patient, with bleeding being the most frequently attributed to medication use [84].

#### 5.5.4. Care Transition Support

The hospital-to-home transition represents a particularly high-risk period for medication errors and care fragmentation. In their study Arima et al. (2025) highlight the essential role of pharmacists in ensuring safe medication transitions, including the use of the simple suspension method for patients with dysphagia, collaborative discharge planning, and education of primary caregivers [85].

A pilot randomized controlled trial by Xu et al. (2025) evaluating a Patient for Medication Safety (PFMS) intervention for older adults with chronic disease during hospital-to-home transition (*n* = 30) found the intervention feasible with an 81.3% adherence rate and no adverse events [86]. Included in the intervention are medication reconciliation, bedside education, a portable pillbox, and six remote motivational interviews. Significant improvements were observed in patient participation in medication safety (time effect, *p* < 0.05), health literacy (between-group effect, *p* < 0.05), and medication discrepancies (time and interaction effects, *p* < 0.05).

#### 5.5.5. Practical Strategies

A stakeholder-informed review by Cai et al. (2025) identified several practical strategies for improving medication management in older adults with dementia, including: (1) secure medication storage to decrease adverse event risk; (2) use of cognitive aids for medication administration; and (3) provision of dementia-specific training to home health staff [34].

#### 5.5.6. Clinical Implications

Multifaced approaches are required to properly address socioeconomic and caregiver barriers, those including: (1) routine screening for financial barriers to medication access; (2) health literacy assessment and tailored education; (3) referral to medication assistance programs for low-income patients; (4) caregiver assessment and support; (5) pharmacist-led medication reconciliation during care transitions; and (6) use of adherence aids such as pillboxes and simplified regimens [64,80,81,85].

**Figure 5:**
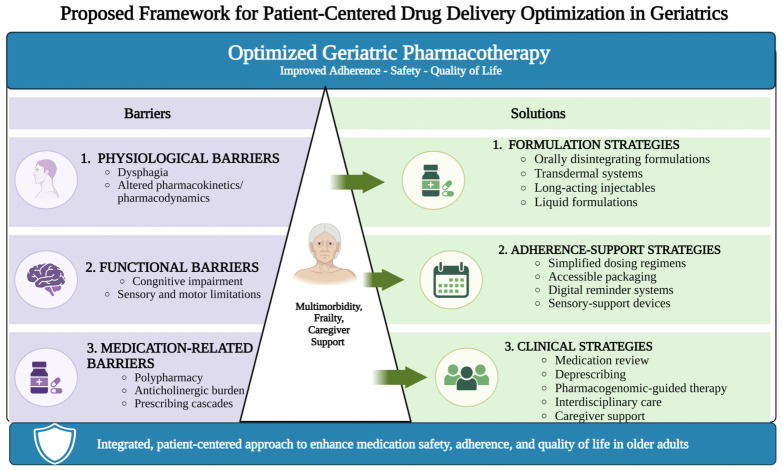
Framework for patient-centered drug-delivery optimization in older adults. Framework summarizing major barriers and patient-centered strategies for optimizing drug delivery and pharmacotherapy in older adults. Created in BioRender. Bania, K. (2026) https://BioRender.com/53aqsik (accessed on 3 May 2026).

Collectively, the barriers affecting drug delivery in older adults are multifactorial and highly interconnected, involving physiological decline, multimorbidity, polypharmacy, cognitive impairment, functional limitations, and social determinants of health. Consequently, optimization of pharmacotherapy in geriatric populations requires integrated patient-centered approaches that combine individualized drug delivery systems, deprescribing strategies, interdisciplinary care, caregiver involvement, and emerging technological solutions. A conceptual framework summarizing the major barriers and patient-centered optimization strategies in geriatric drug delivery is presented in Figure 5.

## 6. Advanced Drug Delivery Systems

The unique physiological, functional, and cognitive challenges faced by older adults have driven the development of innovative drug delivery systems designed to improve medication adherence, safety, and therapeutic outcomes. These systems aim to reduce dosing frequency, simplify administration, bypass barriers to oral delivery, and enable personalized therapeutic approaches [37,41]. Be that as it may, much of the currently available evidence for advanced drug delivery technologies in geriatrics remains preclinical, device-focused, or derived from small observational studies rather than large randomized trials specifically involving frail older populations. Hence, the translational gap between technological innovation and routine clinical implementation remains substantial [37,87].

### 6.1. Modified Oral Formulations

Modified oral formulations address common geriatric barriers such as dysphagia, polypharmacy, and cognitive impairment. Orally disintegrating tablets (ODTs), oral films, and minitablets dissolve rapidly in the oral cavity without the need for water or intact swallowing function. They are particularly useful for patients suffering from Alzheimer’s or Parkinson’s disease who experience swallowing difficulties [67].

### 6.2. Transdermal Delivery Systems

Transdermal delivery systems offer several advantages for geriatric patients, including bypassing gastrointestinal absorption issues, avoiding first-pass metabolism, providing sustained drug release over extended periods, and eliminating the need for swallowing or injection [88].

A 2026 phase III randomized double-blind trial by Nakamura et al. evaluated a novel twice-weekly rivastigmine transdermal patch in 354 patients with mild-to-moderate Alzheimer’s disease [88]. The study demonstrated that the twice-weekly patch was non-inferior to the existing once-daily rivastigmine patch, with analysis suggesting potentially greater efficacy (*p* = 0.032). Adverse events were similar between groups, with application site pruritus being the most common (27.6% vs. 17.1%). The twice-weekly dosing schedule offers greater convenience, which may translate to improved adherence in dementia patients.

Transdermal delivery of gerotherapeutic candidates has also been explored in preclinical trials. As demonstrated by Mao et al. (2025) novel solvent-based topical curcumin formulation delivered twice weekly improved vascular health in hypertensive rats and enhanced multiple heath span indices in aged mice, including improved exercise tolerance and reduced frailty status (*p* < 0.05) [89]. These findings suggest that transdermal delivery may help overcome the poor oral bioavailability commonly associated with natural compounds.

### 6.3. Long-Acting Injectable Formulations

Long-acting injectable (LAI) formulations provide sustained drug release over weeks or months, dramatically reducing dosing frequency. Chronic conditions requiring long-term treatment (e.g., antipsychotic therapy in older adults with schizophrenia or bipolar disorder) require these systems. Reduced dosing frequency improves adherence, decreases caregiver burden, and minimizes medication administration errors [37,41].

### 6.4. Implantable and Depot Systems

Implantable devices offer extended drug release over months to years, making them attractive for chronic age-related conditions. These systems frequently employ biodegradable polymers, hydrogel matrices, osmotic pumps, or bioresponsive biomaterials engineered to achieve sustained and predictable release kinetics. Material selection markedly shapes biodegradation rate, inflammatory response, mechanical stability, and drug diffusion properties. Although several ophthalmologic depot systems have reached advanced clinical development, many implantable geriatric delivery technologies remain limited by procedural invasiveness, manufacturing complexity, device costs, and regulatory approval requirements. Long-term implantation also raises concerns regarding biofilm formation, fibrosis, device failure, and retrieval difficulty in frail patients with multiple comorbidities [74,87].

In ophthalmology, sustained-release drug delivery systems for age-related macular degeneration (AMD) are emerging as promising alternatives to frequent intravitreal injections. Hydrogel-based platforms and implantable devices for long-term ani-VEGF (anti–vascular endothelial growth factor) delivery were reviewed by Seber et al. (2025) and authors reported that these systems can maintain therapeutic drug levels with minimal invasiveness [87]. These systems reduce treatment burden and improve patient quality of life [87,90].

Choe et al. (2025) further reviewed advanced delivery systems for AMD, including hydrogels, nanocarriers, biologically derived vesicles, microneedles, ultrasound-mediated systems, magnetically guided systems, and 3D bioprinted implants [90]. These technologies aim to reduce injection frequency and improve therapeutic outcomes.

### 6.5. Nanotechnology-Based Delivery

Nanotechnology-based drug delivery systems (NDDS) offer precise targeting, controlled release, and enhanced bioavailability—advantages particularly relevant for aging-related diseases. However, many of these approaches remain at preclinical or early translational stages, and their long-term safety, scalability, and applicability in frail geriatric populations have not yet been fully substantiated. Therefore, these emerging technologies should be interpreted as promising investigational strategies rather than fully validated standards of care.

From a mechanistic perspective, these systems exploit nanoscale physicochemical properties such as particle size, surface charge, ligand functionalization, and stimuli-responsive release kinetics to improve drug distribution and cellular uptake. Lipid nanoparticles, polymeric nanoparticles, dendrimers, liposomes, and biomimetic vesicles can be synthesized to protract circulation time, evade immune clearance, and facilitate tissue-specific targeting, including transport across the blood–brain barrier [91,92].

In neurodegenerative disorders, nanoparticle surface modification with peptides, antibodies, or transferrin ligands has been evaluated to increase receptor-mediated transcytosis across the blood–brain barrier. Stimuli-responsive nanocarriers capable of pH-, enzyme-, or reactive oxygen species-triggered release may also allow localized drug activation within pathological microenvironments associated with neuroinflammation and cellular senescence.

Albeit promising preclinical outcomes, major translational barriers remain. Challenges encompass large-scale manufacturing reproducibility, long-term nanomaterial biocompatibility, immunogenicity, regulatory standardization, and limited geriatric-specific safety data. Moreover, relatively few nano-delivery platforms have progressed to late-phase clinical trials in older populations.

Wang et al. (2025) comprehensively reviewed nano-strategies targeting cellular senescence, a primary driver of aging [91,92,93]. NDDS enable senolytic and senomorphic drug delivery, combination therapies, and stimuli-responsive release triggered by pH, reactive oxygen species, or enzyme activity.

In case of neurodegenerative diseases, Gao et al. (2025) reviewed nanoparticle systems capable of crossing the blood–brain barrier (BBB), including polymeric nanoparticles, inorganic nanomaterials, liposomes, and biomimetic carriers [92]. Site-specific drug release is enabled by stimuli-responsive nanocarriers, while organelle-targeting strategies (e.g., mitochondria, lysosomes) provide subcellular precision. Highlighted by Parvin et al. (2025) integration of smart nanomaterials into pharmaceutics for personalized healthcare, emphasizes stimuli-triggered systems for controlled release [93].

### 6.6. Smart Drug Delivery and Digital Health Integration

Digital health technologies are increasingly integrated with drug delivery to optimize medication use in older adults. Contemporary smart drug delivery systems progressively integrate biosensors, wearable devices, cloud-based monitoring platforms, and AI-assisted clinical decision support to enable dynamic and individualized therapeutic adjustment. Mechanistically, these systems are mediated by continuous acquisition of physiological or pharmacological data, followed by algorithm-driven interpretation that can guide dosing recommendations, adherence monitoring, or automated drug release [36].

Closed-loop drug delivery constitutes one of the most advanced forms of this approach. In such systems, real-time physiological feedback (e.g., glucose levels, cardiovascular parameters, or neural activity) is integrated with automated dosing algorithms capable of adjusting therapy with minimal clinician intervention. While closed-loop insulin delivery systems are the most clinically established example, parallel concepts are being actively explored for a range of other conditions, including neurological disorders (e.g., epilepsy and Parkinson’s disease), pain management, and cardiovascular disease [94].

However, operationalization in geriatric populations presents unique challenges, including digital literacy limitations, cognitive impairment, interoperability between healthcare systems, cybersecurity concerns, algorithm transparency, and regulatory oversight of adaptive AI-based systems. Furthermore, most AI-driven medication optimization models have been trained using datasets that underrepresent frail older adults with multimorbidity, potentially limiting external validity and increasing risk of biased recommendations [36].

Vamadevan et al. (2025) systematically reviewed technology-supported medicines optimization interventions for older adults with multimorbidity and polypharmacy [36]. Successful interventions included clinical decision support systems (CDSS), electronic prescribing platforms, pharmacist-led deprescribing systems, and telehealth-supported medication reviews.

Thanks to the NASSS (Non-adoption, Abandonment, Scale-up, Spread, and Sustainability) framework, the review identified key success factors: (1) user-centered technology design; (2) strong clinical leadership; (3) workflow integration; (4) organizational readiness; (5) multidisciplinary collaboration; (6) continuous feedback; and (7) iterative adaptation. Decision tree analysis identified usability, early stakeholder engagement, organizational alignment, and system adaptability as key predictors of successful adoption and sustainability [36].

Another smart delivery approach is represented by the memory incorporating Internet of Things (IoT) technology. Fujita et al. (2026) compared medication management behaviors in healthy older adults and Alzheimer’s disease patients using IoT devices [67]. Operational errors occurred in both groups. Among common success factors were comprehension of spoken language and prospective memory, while disorientation and attention deficits specifically affected the AD group.

Importantly, the current evidence base supporting advanced drug-delivery strategies in geriatric pharmacotherapy remains highly heterogeneous. While certain approaches, including modified oral formulations and transdermal systems, are already integrated into routine clinical practice, many emerging technologies such as AI-assisted dosing platforms, nanotechnology-based carriers, and wearable-integrated delivery systems remain supported primarily by preclinical, feasibility-based, or early translational evidence. Furthermore, frail older adults with multimorbidity and cognitive impairment continue to be underrepresented in large randomized clinical trials, limiting generalizability and certainty of evidence. To provide a clearer translational perspective, the major drug-delivery approaches discussed in this review are summarized according to current level of evidence and implementation status in Table 2.

### 6.7. Translational and Regulatory Challenges

Despite rapid technological progress, translation of advanced drug delivery systems into routine geriatric care remains limited. Several barriers contribute to this implementation gap. First, older adults with frailty, multimorbidity, and cognitive impairment remain underrepresented in clinical trials evaluating advanced delivery technologies, limiting external validity and safety generalizability [102]. Second, regulatory pathways for combination products integrating drugs, devices, biosensors, and adaptive AI algorithms remain complex and incompletely standardized across jurisdictions [103].

Manufacturing scalability, long-term biocompatibility, cybersecurity protection, interoperability with electronic health record systems, and reimbursement uncertainty further complicate implementation. In addition, technology acceptance among older adults may be influenced by digital literacy, caregiver support, and usability considerations. Consequently, many emerging geriatric drug delivery technologies remain at early or intermediate technology readiness levels despite promising proof-of-concept data [41,104,105].

Future development should prioritize pragmatic clinical trials, human-factor engineering, geriatric-specific usability assessment, and regulatory harmonization to facilitate safe translation into routine clinical practice.

## 7. Personalized Medicine and AI

The development of personalized and precision medicine strategies has been motivated by the considerable interindividual variability in drug response observed in older adults. The aim of these approaches is to move beyond traditional “one-size-fits-all” prescribing toward individualized therapy that optimizes efficacy while minimizing toxicity and adverse drug reactions [106,107].

### 7.1. Pharmacogenomics

Pharmacogenomics investigates how genetic variations influence drug metabolism, transport, and response. From a mechanistic perspective, polymorphisms affecting cytochrome P450 enzymes, drug transporters, and receptor signaling pathways may materially alter pharmacokinetics and pharmacodynamics in older adults. Variants involving CYP2D6, CYP2C19, CYP3A4, ABCB1, and SLCO1B1 are particularly relevant in geriatric populations exposed to polypharmacy [106,108].

Despite growing availability of pharmacogenomic testing, real-world implementation remains inconsistent. Major barriers include cost, limited clinician training, uncertain reimbursement pathways, lack of harmonized international guidelines, and insufficient prospective evidence demonstrating cost-effectiveness in multimorbid older populations. In addition, genotype-guided prescribing is complicated by phenoconversion, where drug–drug interactions alter metabolic activity independently of genetic background [109].

In geriatric patients, pharmacogenomic-guided dosing holds particular promise due to the high prevalence of polypharmacy and altered drug handling [108].

#### 7.1.1. Clinical Evidence in Geriatric Populations

A 2025 cross-sectional study by Kondrakhin et al. evaluating 197 older adults (mean age 83 ± 8 years) with non-valvular atrial fibrillation treated with apixaban demonstrated the clinical relevance of pharmacogenomic testing in older adults. The study discovered that carriers of the ABCB1 rs1045642 CC genotype were at significantly higher risk of haemorrhagic complications compared to alternative alleles (OR = 2.805; 95% CI: 1.326–5.935; *p* = 0.006), with the association remaining significant after correction for multiple testing under the log-additive model (OR = 1.93 per C allele; 95% CI: 1.17–3.20; q = 0.0275). Concomitant therapy, particularly with antiarrhythmic drugs and statins (rosuvastatin), also increased bleeding risk, underscoring the importance of considering both genetic and drug–drug interaction factors [106].

#### 7.1.2. Phenoconversion in Multimedicated Geriatric Patients

Phenoconversion, which is a mismatch between genotype-predicted and actual drug–drug metabolizing phenotype caused by drug–drug interactions poses a significant challenge in geriatric pharmacogenomics. A 2025 observational study by Sarömba et al. addressed this issue using solanidine metabolites (SSDA and 4-OH-solanidine) as diet-derived CYP2D6 biomarkers in 88 geriatric patients (median age 83 years, median 15 medications). Genotype-derived activity scores (*p* < 0.001) correlated significantly with the SSDA/solanidine metabolic ratio and clearly detected poor metabolizers. In a model adjusted for age, sex, Charlson Comorbidity Index, and estimated glomerular filtration rate, each additional CYP2D6 substrate/inhibitor significantly lowered the expected activity score by 0.53 points (95% CI: 0.85–0.21) in patients encoding functional CYP2D6 variants (R^2^ = 0.242). These show that phenotyping using diet-derived biomarkers can elucidate phenoconversion in geriatric patients, and serve as a prerequisite for personalized drug prescribing in multimorbid populations [109].

### 7.2. Biomarker-Guided Drug Delivery

Biomarkers (measurable indicators of biological processes or pharmacological responses) are increasingly used to guide drug selection, dosing, and monitoring in older adults.

#### 7.2.1. Neurodegenerative Disease Biomarkers

A 2026 phase III clinical trial by Wischik et al. evaluating the tau aggregation inhibitor hydromethylthionine mesylate (HMTM) in 598 amyloid β-PET positive participants with mild cognitive impairment (MCI) and mild-to-moderate Alzheimer’s disease incorporated multiple biomarker outcomes. The article demonstrated a reduction in the progression of neurodegeneration measured by neurofilament light chain (NfL) change at 52 weeks in the whole population, consistent with significant reductions in progression of grey matter atrophy at 52 and 104 weeks, and a reduction in progression of tau pathology (pTau217, *p* = 0.0165) in MCI participants. Clinical outcome measures were complimented due to these biomarker findings, which provided objective evidence of target engagement and disease-modifying effects [110].

#### 7.2.2. Vascular Aging Biomarkers

When reviewing the current state of vascular aging pharmacology, Zheng et al. (2026) identified key pathogenic mechanisms including: (1) epigenetic drift; (2) chronic low-grade inflammation; and (3) cellular senescence, with clinically relevant targets such as IL-1β pathway and senescent cell populations [111]. The need for validated surrogate endpoints and liquid biopsy was emphasized by the authors to accelerate clinical trials of therapies promoting vascular health resilience.

### 7.3. Therapeutic Drug Monitoring

Therapeutic drug monitoring (TMD) is a measurement of drug concentration to individualize dosing and is particularly valuable in older adults, where altered pharmacokinetics and polypharmacy create substantial variability in drug exposure.

#### 7.3.1. Model-Informed Precision Dosing

A review by Le et al. (2026) introduced model-informed precision dosing (MIPD) as a conceptual framework integrating therapeutic drug monitoring with machine learning (ML) and artificial intelligence (AI) within population health informatics [112]. Traditional TMD is limited by manual interpretation and specific constraints such as sampling at steady-state and requiring a minimum of two drug concentrations. MIPD with the Bayesian method represents a scalable innovation that combines AI/ML with comprehensive electronic health records to offer real-time, precise dosing adjustments. This integration presents a potential to improve patient safety, reduce health costs, and optimize therapeutic outcomes, predominantly for vulnerable populations including geriatric patients where evidence is limited.

#### 7.3.2. Closed-Loop Medication Safety Management

An observational study conducted by Chen et al. (2026) evaluated a closed-loop medication safety management systems among 100 older inpatients with rheumatic and immunologic diseases [113]. Included in the intervention were participation of clinical pharmacists, risk alerts through digital systems, and full-process medication tracking. After the intervention, the potentially inappropriate medication (PIM) incidence in the intervention group dropped notably to 16%, which is markedly lower than the 58% in the control group (*p* < 0.05). The rate of appropriate prescribing increased to 86% in the intervention group versus 44% in the control group (*p* < 0.05). The incidence of adverse drug reactions decreased from 24% to 8% in the intervention group (*p* < 0.05), while medication adherence rose to 90% and patient satisfaction reached 96%. This study shows that medication safety closed-loop management—using the deep integration of information technology and multidisciplinary collaboration—significantly improves medication safety and appropriateness in older adults.

### 7.4. AI-Based Dose Optimization

AI and ML are increasingly applied in order to optimize medication regimens in older adults, addressing the complexity of polypharmacy and multimorbidity.

#### 7.4.1. AI in Polypharmacy Management

A 2025 scoping review by Bringhurst et al. assessed AI applications in managing multiple pharmacological therapies among adults aged 50 and older. The authors describe that AI tools demonstrated efficiency and accuracy eliminating drug–drug interactions, assisting in the detection of potentially inappropriate medications, and identifying patterns of multimorbidity because of polypharmacy in older adults. In addition, AI tools were considered easy to use and helped enhance medication adherence. Based on the results AI has potential in managing polypharmacy, particularly in enhancing medication safety, improving adherence, and predicting risk factors for medication-related errors in older adults [114].

#### 7.4.2. AI-Enabled Precision Medicine for Multimorbidity

A 2026 review by Deng et al. examined the future of multimorbidity management in older adults through AI-enabled precision medicine, proposing a novel autonomy-based framework within the extended Healthcare 5.0 architecture. AI applications are classified across four autonomy tiers–from clinician-guided tools to adaptive, agentic systems–highlighting how each can support intervention optimization, patient stratification, and care personalization in older populations. Among key AI technologies are machine learning and deep learning for comorbidity prediction (achieving high accuracy in predicting chronic disease comorbidities), natural language processing for extracting structural information from unstructured medical text, and computer vision for medical image analysis in multimorbidity management. The authors put emphasis on how AI-powered personalized medicine can improve effectiveness and increase patient adherence compared to traditional approaches, though challenges remain in data integration, algorithm interpretability, and ethical deployment [107].

#### 7.4.3. Clinical Implementation Considerations

**Figure 6:**
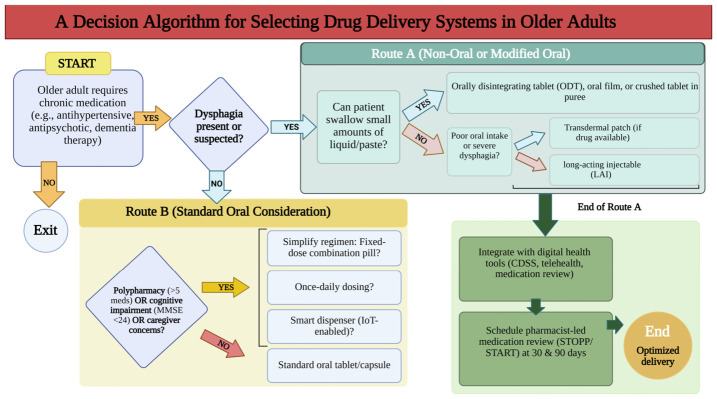
Clinical decision algorithm for the selection of advanced drug delivery systems in geriatric care. Simplified conceptual algorithm for selecting appropriate drug-delivery strategies in older adults based on functional and clinical factors. Created in BioRender. Bania, K. (2026) https://BioRender.com/ghl449n (accessed on 4 May 2026).

Peterson et al. (2026) reviewed strategies for identifying and resolving drug-related problems in geriatric patients, emphasizing the need for routine drug assessment within multidisciplinary teams across the care continuum [108]. Fit for the Aged criteria, Medication Appropriateness Index, American Geriatric Society Beers Criteria, and STOPP/START criteria (Screening Tool of Older Persons’ Prescriptions and Screening Tool to Alert to Right Treatment) are some of the key tools included in the medication selection in older adults. Drug therapy problems must be identified through medication reconciliations performed at admission, discharge, and during transitions of care, while physicians and advanced practice providers must routinely review medication lists, deprescribe when able, and prevent the prescribing cascade (Figure 6).

## 8. Future Perspectives and Challenges

Despite significant innovation in drug delivery systems, few technologies have been widely used in geriatric medicine. Some available methods, like transdermal systems, long-acting injectable formulations, automated insulin delivery platforms, and certain implantable eye devices, have shown they can work in clinical settings and have received regulatory approval. In contrast, many systems based on nanotechnology, AI-driven dosing platforms, biomaterial-responsive carriers, and multifunctional theranostic systems are still in preclinical or early stages of development [92,93].

Translation from experimental proof-of-concept to routine clinical use is limited by several connected barriers. Manufacturing scalability and batch reproducibility are major challenges for nanomedicine platforms, especially for complex multifunctional carriers that need precise physicochemical control. Long-term safety regarding biocompatibility, immunogenicity, and degradation is not fully understood in frail older populations with multimorbidity and changed physiology [91,92].

Economic feasibility is another major concern. Many advanced delivery technologies need expensive manufacturing processes, digital infrastructure integration, specialized monitoring systems, and multidisciplinary implementation plans. As a result, cost-effectiveness data are still limited, especially in publicly funded healthcare systems that are already facing challenges from an aging population and increasing chronic disease rates [115].

### 8.1. Advancing Drug Delivery Technologies

A promising frontier for geriatric pharmacotherapy (particularly for chronic age-related conditions) is represented by the development of sustained-release drug delivery systems. In ophthalmology, sustained-release systems for age-related macular degeneration—including hydrogel-based platforms and implantable devices—offer the potential to maintain therapeutic drug levels with minimal invasiveness, and reduce the burden of frequent intravitreal injections. Nevertheless, challenges in manufacturing complexity, long-term safety, and regulatory approval must be addressed before these technologies can be widely adopted [87].

Advanced delivery systems for age-related macular degeneration treatment continue to evolve, encompassing hydrogels, nanocarriers, biologically derived vesicles, microneedles, ultrasound-mediated systems, magnetically guided systems, 3D bioprinting, and implantable sustained-release devices. While the aim of these technologies is to reduce injection frequency and improve therapeutic outcomes, the clinical adoption of these innovative therapies requires further evidence on patient safety, quality of life, and efficacy [89].

When it comes to neurodegeneration, nanotechnology-based drug delivery systems targeting cellular senescence represent a transformative approach. Thanks to those systems, senolytic and senomorphic drug delivery is enabled, along with combination therapies and stimuli-responsive release mechanisms that can be triggered by pH, reactive oxygen species (ROS), or enzyme activity [90]. For central nervous system (CNS) disorders, intrathecal administration of therapeutic directly into cerebrospinal fluid (CSF) bypasses blood–brain barrier (BBB), offering promising avenues for antisense oligonucleotides, antibodies, stem cells, and gene therapies. However, the potency of these novel treatments is often curtailed by the limitations of first-generation delivery systems, which struggle with uneven distribution, systemic leakage, and the demands of moder biologics [116].

### 8.2. Leveraging Artificial Intelligence

AI has demonstrated significant potential in terms of medication management for older adults, specifically in eliminating drug–drug interactions, detecting potentially inappropriate medications, and enhancing medication adherence. Within the Veterans Health Administration, AI technologies (including natural language processing, ML, large language models, clinical decision support, and data analytics) offer opportunities to increase the evidence-based implementation of the Age-Friendly Health Systems 4Ms framework [117].

In low- and middle-income countries, AI adoption remains limited despite its benefits due to high implementation costs, insufficient digital infrastructure, low AI literacy among healthcare providers, and ethical concerns related to data privacy and algorithm bias. In order to address these barriers, strategic policy reforms, investment in AI education, and improved regulatory frameworks are required to ensure responsible and equitable AI deployment. Future research should be focusing on evaluating the long-term effectiveness of AI interventions in real-world settings and developing scalable solutions tailored to low- and middle-income countries, where the aging transition is occurring most rapidly [118].

### 8.3. Digital Health Integration

Information and communication technologies designed to support chronic disease self-management among older people have grown rapidly, targeting behaviors such as medication adherence, physical activity, dietary management, and follow-up care. The current evidence base remains fragmented, with substantial variability in technology types, targeted behaviors, and reported outcomes. There are several gaps remaining in regard to the participatory design of these technologies with older adults and the development of technologies that address multiple self-management need simultaneously. Future technology development should intentionally incorporate older adults and caregivers throughout the design cycle and expand beyond single-behavior interventions to reflect the multimorbidity common in this population [119].

The major translational barriers, implementation challenges, evidence gaps, and future research priorities associated with advanced drug-delivery strategies in geriatric pharmacotherapy are summarized in Table 3.

## 9. Conclusions

Optimizing drug delivery in older adults has become a major challenge in modern healthcare due to rapid population aging and the increasing prevalence of multimorbidity, frailty, and polypharmacy. Age-related physiological changes that influence pharmacokinetics and pharmacodynamics significantly complicate medication management and contribute to increased susceptibility to adverse effects.

Traditional, universal pharmacotherapy methods often prove insufficient in geriatric populations. Consequently, there is a growing need for personalized, patient-centered drug delivery strategies that address the complex clinical realities of older adults. New approaches—including modified oral formulations, transdermal patches, long-acting injectable therapies, nanotechnology-based platforms, pharmacogenomics-guided dosing, and AI-assisted therapeutic optimization—show significant potential to improve medication adherence, safety, and therapeutic efficacy. At the same time, optimized geriatric pharmacotherapy requires more than just technological innovation. Structured medication review, tapering strategies, care models, and active patient and caregiver engagement remain essential components of safe and effective pharmacological treatment for older adults. Existing scientific evidence supports the clinical importance of reducing medication burden and minimizing potentially inappropriate prescribing.

Despite significant advances, many new drug delivery technologies are still primarily based on observational, translational evidence, while older adults with multimorbidity remain underrepresented in clinical trials. Future research should therefore prioritize geriatric research, standardized methodological frameworks, clinically relevant outcomes, and implementation-oriented studies that can translate innovations into current clinical practice.

Optimizing medication delivery for geriatric patients requires a shift toward flexible, personalized, and interdisciplinary care models that address the physiological, functional, and social complexities of aging populations.

## Figures and Tables

**Table 1:** Age-related pharmacokinetic changes and therapeutic implications in older adults.

Pharmacokinetic Process	Age-Related Physiological Change	Clinical Consequence	Therapeutic Implication
Absorption	Reduced gastric acid secretion, delayed gastric emptying, decreased gastrointestinal motility	Altered absorption kinetics of selected medications	Variable oral bioavailability and delayed onset of action
Distribution	Increased adipose tissue, reduced total body water, decreased lean body mass, reduced serum albumin	Altered volume of distribution and protein binding	Prolonged half-life of lipophilic drugs and increased free fraction of highly protein-bound medications
Metabolism	Reduced hepatic blood flow and decreased hepatic enzymatic activity	Slower drug biotransformation and clearance	Increased risk of drug accumulation and adverse effects
Excretion	Decline in renal blood flow, glomerular filtration rate, and nephron function	Reduced elimination of renally cleared drugs	Requirement for dose adjustment and renal function monitoring

**Table 2:** Current level of evidence and translational maturity of advanced drug-delivery strategies in geriatric pharmacotherapy.

Drug-Delivery Strategy	Current Evidence Base	Clinical Implementation Status	Major Limitations	References
Orally disintegrating tablets and modified oral formulations	Supported by clinical studies and established use in dysphagia management in older adults	Established clinical approach	Limited personalization; adherence still dependent on cognition and caregiver support	[95]
Transdermal delivery systems	Moderate clinical evidence supporting chronic disease management and sustained drug release	Widely implemented for selected medications	Variable skin absorption, age-related skin fragility, irritation risk	[96]
Long-acting injectable systems	Increasing clinical evidence for adherence improvement and reduced dosing frequency	Clinically available for selected therapeutic areas	Limited geriatric-specific randomized evidence and monitoring requirements	[97]
Pharmacogenomic-guided dosing	Supported by growing pharmacogenetic and observational evidence	Partial clinical implementation	Limited access, cost, inconsistent external validation in frail older adults	[98]
AI-assisted dosing and clinical decision-support systems	Primarily feasibility-based and early-stage clinical evidence	Emerging technology	Algorithmic bias, interoperability barriers, limited validation in multimorbid geriatric populations	[99]
Nanotechnology-based drug-delivery systems	Predominantly preclinical and translational research	Experimental	Regulatory uncertainty, scalability challenges, limited long-term safety data	[100]
Smart wearable-integrated and closed-loop delivery systems	Early-stage translational and prototype-based evidence	Mostly experimental	High cost, technological complexity, limited real-world geriatric implementation	[101]

**Table 3:** Translational readiness, implementation barriers, and future research priorities for advanced drug-delivery strategies in geriatric patients.

Technology/Strategy	Current Clinical Status	Major Limitations/ Evidence Gaps	Future Priorities	References
Transdermal systems	Widely available; high translational readiness	Interindividual variability in skin permeability and absorption	Optimization for frail older adults and multimorbidity-specific dosing strategies	[37,87]
Long-acting injectable systems	Clinically available with established efficacy in selected indications	Cost, adherence monitoring, and limited geriatric-specific evidence	Evaluation of long-term outcomes and broader geriatric implementation	[87,90]
Smart pill dispensers and digital adherence tools	Limited clinical implementation	Digital literacy barriers, usability concerns, limited long-term effectiveness data	Development of user-centered interfaces and real-world implementation studies	[36]
Closed-loop and AI-assisted dosing systems	Early-to-moderate implementation	Algorithm validation, bias, regulatory uncertainty, interoperability issues	Large-scale validation studies and integration into geriatric care pathways	[36,94]
Nanocarrier-based delivery systems	Primarily experimental/preclinical	Scalability, toxicity concerns, manufacturing complexity	Long-term safety trials and translational standardization	[93,103]
Theranostic and precision medicine platforms	Experimental with limited clinical application	Regulatory complexity, insufficient geriatric-specific evidence	Integration of pharmacogenomics, biomarkers, and personalized therapeutic algorithms	[92,93]
Deprescribing and medication optimization frameworks	Increasing clinical adoption	Heterogeneity of evidence, implementation variability, lack of standardized outcomes	Pragmatic RCTs, implementation science approaches, and patient-centered deprescribing models	[117,119,120]
Clinical practice guidelines for multimorbidity and polypharmacy	Available but methodologically heterogeneous	Limited feasibility, insufficient patient-centered integration	Improved methodological rigor and incorporation of patient and caregiver perspectives	[32,87,90]

## Data Availability

No new data were created or analyzed in this study. Data sharing is not applicable to this article.
